# Exploring Spin Distribution and Electronic Properties in FeN_4_-Graphene Catalysts with Edge Terminations

**DOI:** 10.3390/molecules29020479

**Published:** 2024-01-18

**Authors:** Ismail Can Oguz, Frederic Jaouen, Tzonka Mineva

**Affiliations:** ICGM, Univ. Montpellier, 34293 Montpellier, France; i.c.oguz@differ.nl (I.C.O.); frederic.jaouen@umontpellier.fr (F.J.)

**Keywords:** FeN_4_-C catalysts, spin, band structure, zigzag graphene nanoribbons, armchair graphene nanoribbons, DFT

## Abstract

Understanding the spin distribution in FeN_4_-doped graphene nanoribbons with zigzag and armchair terminations is crucial for tuning the electronic properties of graphene-supported non-platinum catalysts. Since the spin-polarized carbon and iron electronic states may act together to change the electronic properties of the doped graphene, we provide in this work a systematic evaluation using a periodic density-functional theory-based method of the variation of spin-moment distribution and electronic properties with the position and orientation of the FeN_4_ defects, and the edge terminations of the graphene nanoribbons. Antiferromagnetic and ferromagnetic spin ordering of the zigzag edges were considered. We reveal that the electronic structures in both zigzag and armchair geometries are very sensitive to the location of FeN_4_ defects, changing from semi-conducting (in-plane defect location) to half-metallic (at-edge defect location). The introduction of FeN_4_ defects at edge positions cancels the known dependence of the magnetic and electronic proper-ties of undoped graphene nanoribbons on their edge geometries. The implications of the reported results for catalysis are also discussed in view of the presented electronic and magnetic properties.

## 1. Introduction

The past two decades have seen significant attention directed toward controlling electronic spin moments in graphene nanostructures. It began with the theoretical prediction of spin-polarizable mono-hydrogenated edge states in zigzag graphene nanoribbons (ZGNRs) [1], paving the way for creating magnetic graphene finite-size structures. These structures hold promise for applications in spintronics and quantum information technologies. However, synthesizing magnetic monohydrated ZGNRs remains challenging [2,3], leading to extensive theoretical [3,4,5,6,7,8,9,10,11] and experimental efforts [12,13,14,15,16,17,18]. The objective is to understand, modify, and control the spin-density distributions in nanostructured graphene. Among the strategies for shaping graphene’s magnetic properties, doping with heteroatoms has emerged as a general method for tuning magnetic, electronic, optical, and transport properties [9,18,19,20,21,22,23]. Computational studies using density functional theory (DFT) revealed that doping GNRs with nitrogen, boron, or transition metal (TM) atoms at specific positions can induce thermodynamically stable ferromagnetic GNRs with spin-polarized edge carbons, depending on the edge geometry (zigzag or armchair), ribbon widths, location, and density of dopant atoms [9,20,21,24,25,26,27]. These studies also agree that GNRs with dopants at the edges are generally more energetically stable than those doped far from the edge (in-plane) of the graphene nanoribbons.

The ability of graphene to stabilize various dopants is also used in catalysis by employing graphene to host a variety of active sites, including non-metal dopants and a broad range of single metal atoms, from 3d transition metals to heavier elements, such as Sn and platinum-group metals [28,29,30]. In particular, co-doped graphene with transition metals and nitrogen has been emerging as a promising electrocatalyst for oxygen reduction reactions (ORR) [22,31,32,33], CO_2_ conversion [34,35,36], or nitrate reductions [36]. TMNx-graphene has been identified as an efficient catalyst because of its spin polarization [36,37]. In particular, FeN_4_ moieties that are covalently integrated in carbonaceous matrices have been established as the most promising precious-metal-free active sites in proton exchange membrane fuel cell cathodes for ORR [22,32,38,39,40]. Unrevealing the origins behind the good reactivity of Fe-N-C catalysts has become an important milestone for a more rational design of non-precious metal catalysts. The FeN_x_C_y_ coordination structures and iron, magnetic, and electronic properties are among the key factors that determine the reactivity of Fe-N-C catalysts. Experimental techniques, such as extended X-ray absorption fine structure (EXAFS), X-ray absorption near-edge structure (XANES) spectroscopy, X-ray photoelectron spectroscopy (XPS), and Mössbauer spectroscopy, are commonly used to discern the geometrical, electronic, and magnetic properties of synthesized Fe-N-C catalysts. Meanwhile, DFT calculations applied to truncated or extended FeN_4_-graphene models serve to provide insights into the local iron environment and its electronic structure [32,41,42,43,44,45,46].

The pyridinic (FeN_4_C_10_) and porphyrinic (FeN_4_C_12_) configurations are widely recognized as the predominant coordination geometries of the active sites in pyrolyzed Fe-N-C catalysts for the oxygen reduction reaction (ORR). By analyzing the computed and measured Mössbauer quadrupole splitting data, the presence of high-spin Fe(III)-N_4_C_12_ porphyrinic structures and low- or medium-spin Fe(II)-N_4_C_10_ pyridinic structures in pyrolyzed Fe-N-C catalysts has been established [42]. DFT studies have revealed the thermodynamic stability of spin-polarized at-edge TM-C_x_N_y_ defects [21,22,31,37,47]. A high magnetic moment computed in FeN_4_-AGNR and FeN_4_-ZGNR was attributed to parallel spin moments on the localized 3d electrons of Fe atom and 2p electrons of C atoms [47]. More recent theoretical studies have correlated the ORR activity with the iron magnetic moments in FeN_4_-graphene, owing to the hybridization between spin-polarized Fe3d and O2p orbitals in the activated OH complex [22]. The enhancement in the performance of the FeN_4_-graphene catalyst was attributed to an alteration in FeN_4_-graphene band structure from metallic to half-metallic [48], meaning that the spin-up bands have isolating character, whereas spin-down bands have conducting character, or vice versa. In light of these studies, the spin distribution in FeN_4_-graphene structures appears to play a pivotal role in their electrocatalytic activity; however, the concomitant spin polarization of graphene edge carbons, possibly occurring in truncated FeN_4_-graphene models, has been considered in very few cases [47]. Comprehending the spin interactions between co-doped iron–nitrogen states and carbon edge states remains an open question, yet it is crucial for regulating the electronic properties and spin density at metal active sites within Fe-N-C materials, particularly for their application as electrocatalysts.

In this study, we examine the spin distribution between iron and carbon atoms, and the sub-band profile of both Zigzag Graphene Nanoribbons and Armchair Graphene Nanoribbons. These ribbons integrate pyridinic-like FeN_4_ defects positioned at various locations between the ribbon’s center and one of its edges. This study explores both ferromagnetic and antiferromagnetic spin ordering at the edges concerning the FeN_4_-GNR’s thermodynamic stability, and electronic and magnetic properties.

## 2. Results

### 2.1. Formation and Stability of Fe(II)N_4_ in ZGNR and AGNR Models

**Figure 1:**
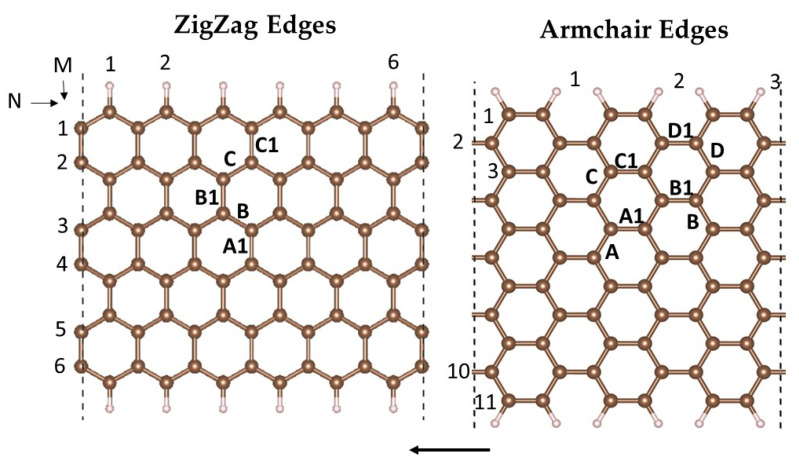
Atomic structure of zigzag (**left**) and armchair (**right**) graphene nanoribbons. The labelled carbon bonds indicate the positions of the vacancies, created by removing two bound carbons. FeN_4_ active sites are subsequently integrated into the double vacancies (see text). The arrows indicate the periodicity direction of ZGNRs and AGNRs. The N and M indicate, respectively, the number of edge atoms and the width of GNR. The atom color code is the following: brown for carbon and white for hydrogen.

Initially, we concentrated on the stability of the pyridinic Fe(II)N_4_ active site, ranging from bulk sites to edge configurations in the graphene nanoribbons with zigzag and armchair terminations, shown in Figure 1, on the left and right sides, respectively. The dangling bonds of the edge carbon atoms are saturated with hydrogen (H).

Conventionally, ZGNRs are described by the number of atoms along a row and the width of the model, denoted by the notation (N × M). Here, ‘N’ represents the number of atoms on the edge row, and ‘M’ indicates the width of the model. The notation we employed was first introduced by Cervantes-Sodi et al. [49]. According to this notation, the ZGNR model shown in Figure 1 was defined as a (6 × 6) ZGNR, and the AGNR model was designated as an (3 × 11) AGNR. To prevent interactions between an Fe atom and its periodic image, graphene nanoribbon models were selected with sufficient size: the distance between the Fe atoms and their periodic images in the adjacent cell was maintained at 14.83 Å for zigzag and 12.83 Å for armchair configurations.

The FeN_4_-GNR unit cells were constructed after the formation of a double vacancy (DV), by subtracting a pair of C atoms and replacing the four dangling C atoms with nitrogens. The Fe atom was then embedded in the DV center, coordinated to four N atoms. We explored five FeN_4_-ZGNR and eight FeN_4_-AGNR positions. The DVs were chosen to represent two different orientations of FeN_4_ with respect to the periodicity axis: orientations perpendicular or aligned with the periodicity axis are labelled as A1, B1, C1, and D1 in Figure 1, hereafter called “direct”; and orientations tilted at 30° or 60° to the ZGNR and AGNR periodicity axes are labelled as A, B, C, and D in Figure 1, hereafter called “tilted”. The energetic and magnetic properties of bare or single-atom-doped GNRs may vary with the ribbon width. Strong dependence of dopant binding and formation energies were reported for very thin ribbons with width M = 2 carbons in the row, but going beyond this limit, the effect of the ribbon widths decreased rapidly. The width of the models considered here (M = 6 for ZGNR and M = 11 for AGNR) is therefore sufficient to exclude a significant variation of the computed properties with variations of the ribbon widths.

**Figure 2:**
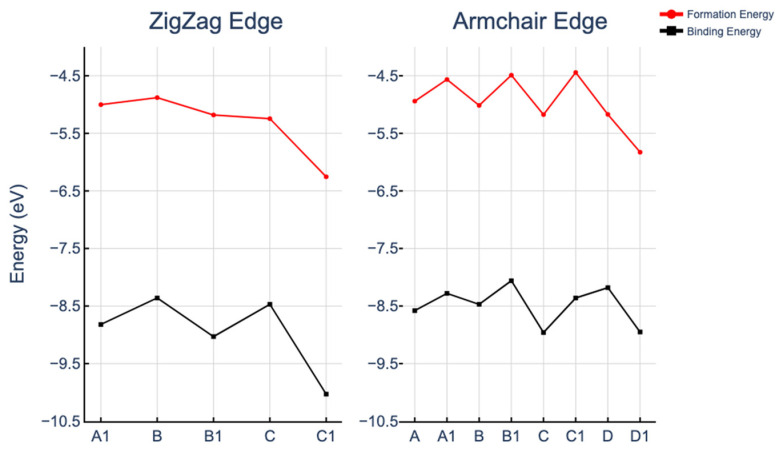
Formation and binding energies of FeN_4_ moieties in ZGNR (**left**) and AGNR (**right**).

The reported formation energies (*E*_f_) and binding energies (*E*_b_) displayed in Figure 2 confirm the thermodynamically stable integration of FeN_4_ defects at all thirteen examined positions within both GNRs. This aligns with the previously established good thermodynamic stability of covalently integrated FeN_4_ moieties in different models of graphene nanostructures [17,18,19,22,44]. Note that, in our comparative study, *E*_f_ and *E*_b_ were computed for the lowest energy structures, whose edge spin configurations were with a ferromagnetic (FM) order. The translocation of the Fe center towards the edge results in the suppression of spin on one GNR side, thereby rendering the antiferromagnetic (AFM) order at the edge untenable. Consequently, to maintain a uniform standard across all models, configurations derived from ferromagnetic ordering at the edges were used to ascertain energy stability. The A1 configuration is an exception because the antiferromagnetic spin orientations of the edges were found to be more stable by 23 meV than the ferromagnetic spin ordering. The definitions used to compute *E*_f_ and *E*_b_ are reported in the Methods section.

The formation energies associated with the FeN_4_ defect varied depending on its orientation. In the case of ZGNRs, the “tilted” orientations (points B and C in Figure 2, left-hand side) were revealed to be less stable than the defects with “direct” orientations (points B1 and C1 in Figure 2, left-hand side). For the armchair configurations, “tilted” orientations generally exhibited greater stability than their “direct” counterparts (compare A, B, and C with A1, B1, and C1 in Figure 2, right-hand side). The only exception to this trend was the stronger stability of the “direct” FeN_4_ in D1 position (at the edge) in comparison with the “tilted” defect in position D (D1 and D points in Figure 2, right-hand side). This can be associated with the different FeN_4_ environments in the D and D1 locations, which apparently contributed more strongly to the FeN_4_ stabilization than the specific orientation of FeN_4_. In the D-placed defect with “tilted” orientation, one nitrogen became pyrrolic-like (at the edge) and the other three nitrogens remained pyridinic, whereas in the D1-placed defect, two nitrogens were pyrrolic-like. The D1 defect had the same carbon environment as the most stable C1 defect in ZGNR, with two pyridinic and two pyrrolic-like nitrogens. Thus, increasing the number of pyrrolic-like nitrogen atoms at the edges increased the thermodynamic stability of FeN_4_-GNRs. This aligns with Li et al.’s findings [27], where nitrogen defects near the edges of ZGNRs were energetically favorable, and pyrrole-like defects possessed even lower formation energies than pyridinic-like defects. Our findings on Fe-centered defect stabilization in graphene nanoribbons are consistent with Holby et al.’s observation [28] of preferential edge stabilization of Fe-pyridinic vacancy complexes (FeN_3_) in GNRs.

Overall, a relatively small variation (within 15%) in the formation and binding energies of FeN_4_ from in-plane positions A and A1 to the edge positions C1 and D1 was obtained. Fluctuations in formation energy were a recurrent theme in the study of nitrogen or metal-substituted graphene nanoribbons [27,28,50,51], underscoring the edge configuration as the most stable site. These investigations have also considered pyridine-like structures, such as 3NV and 4N defects, as in our work, which also demonstrates the highest stability at the edges of zigzag graphene nanoribbons.

### 2.2. Ferromagnetic and Antiferromagnetic Spin Ordering at the ZGNR Edges

The ferromagnetic (FM) and antiferromagnetic (AFM) spin orderings were considered for the minimum energy ZGNRs. For comparison, the magnetic and electronic features of the pristine ZGNR and AGNR were computed at the same theoretical basis. It was generally reported from the DFT calculations of ZGNRs that the FM ordering of the spin-polarized orbitals of the edge carbons was slightly less stable than the AFM phase. Our results are in line with this finding, reporting AFM ordering as more stable by 59 meV than the FM spin orientation. The average spin moment localized at every carbon atom at the zigzag edges was 0.23 ${\;\mu }_{B}$ (Figure S1a). The FM band structure in Figure S1b shows a metallic character because of the crossing of spin-up and spin-down states at the Fermi level. In this band diagram, the spin-up states are shifted to the conduction band and the spin-down states are dominating in the valence band zone. Consistent with earlier findings [1,3,4,7,9,10], the AFM structure (refer to Figure S1c) demonstrated a semiconductor-like nature, displaying a band gap of 0.54 eV with degenerate spin-up and spin-down channels that overlapped. At each edge carbon, the magnetic moment was ±0.24 $\mu_{B}$ , resembling closely those in the FM ZGNR. Analysis of band-decomposed charge density plots in Figure S2 revealed that the bands near the Fermi level were primarily composed of spin-polarized p_z_ orbitals originating from the edge carbons.

**Figure 3:**
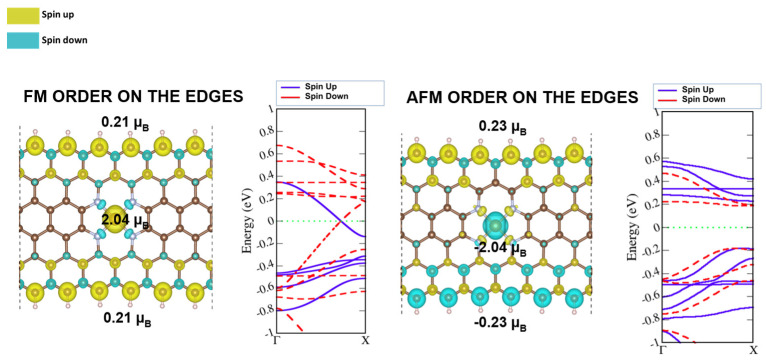
Spin density and band diagram of FM- (**left**) and AFM-ordered (**right**) carbon edges in FeN4-ZGNRs. Yellow and turquoise colors represent the spin-up and spin-down distribution over lattice structure. Blue and dashed red colors represent the spin-up and spin-down states in band diagram.

Fe(II)N_4_ defects located at the central DV (position A1 in Figure 1) in ZGNR decreased negligibly the spin moments at the edge carbons with respect to the pristine GNRs, as revealed by the results in Figure 3, left- and right-hand sides for the FM and AFM ordering, respectively. The absolute value of the spin moment at the iron atom was 2.04 $\mu_{B}$ , for both the AFM and FM ordering of the carbon atoms at the edges (Figure 3). However, the overall AFM-FeN_4_-ZGNR structure was energetically more stable by 23 meV compared with FM-FeN_4_--ZGNR. The total magnetic moment of FeN_4_-ZGNRs therefore resulted from the Fe3d and C p_z_ spin-polarized states and became 4.54 and 2.0 $\mu_{B}$ , respectively, for FM and AFM ordering of the edges.

Despite the FeN_4_ integration into graphene, the band structures preserved their character as in the pristine ZGNR (Figure S1). The FM edges determined a conducting band (Figure 3, left-hand side) and the AFM edges determined a semi-conducting band structure (Figure 3, right-hand side). Doping with FeN_4_ at the in-plane position in ZGNRs did not affect the electronic structure and the magnetic distribution on edge carbons, but only the total magnetic moment of the ribbon.

### 2.3. Magnetic and Electronic Properties as a Function of FeN_4_ Location and Edge Termination

The evolution of the spin-density and sub-band structures of FeN_4_ with different orientations and positions was explored considering the more stable AFM-ZGNR, with opposite spin-moment orientations at the edges and AGNR with non-magnetic (with zero spin moments) edges.

**Figure 4:**
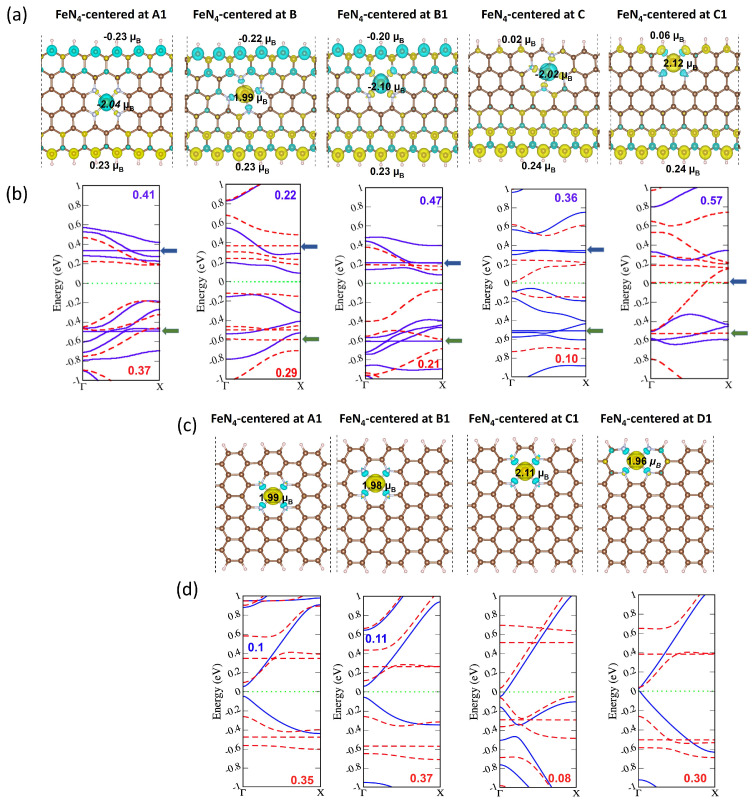
Spin-density and sub-band diagrams of FeN_4_-ZGNRs in (**a**) and (**b**), respectively, and FeN_4_-AGNR in (**c**) and (**d**), respectively. The band-gap energies for spin-down (red dash curves) and spin-up (blue solid curves) channels are in eV. In (**b**), the blue arrows point to the $d_{z^{2}}$ states and the green arrows point to the $d_{{x^{2}-y}^{2}}$ (“direct” orientation) or $d_{xy}$ (“tilted” orientation) states. The Fermi energy is set to zero. Yellow and turquoise colors represent the spin-up and spin-down distribution over lattice structure.

The computed spin-density distributions and band diagrams for the five positions of the FeN_4_ in ZGNRs are presented in Figure 4a,b and those of the four “direct” positions FeN_4_-AGNRs are presented in Figure 4c,d. The band-gap values presented in Figure 4b,d were calculated using the GGA-PBE functional, known to underestimate band gaps for systems with strong electronic correlations. However, while PBE may not yield precise absolute band-gap values, it reliably captures trends in the band structures variations with FeN_4_ location and orientation.

The analysis focused first on zigzag edge models. To prevent miscalculation, adjustments were made to the unit cell structure, specifically due to the FeN_4_ moiety in graphene, considering potential strain-induced modifications in spin moments and edge direction that can influence the transition from antiferromagnetic (AFM) to ferromagnetic (FM) ordering. To mitigate strain effects, a two-step approach was employed. Initially, geometric cells of zigzag models were relaxed for unit cell adjustment, seeking an optimal *x*-axis cell parameter. The final calculation used a fixed cell with the determined unit cell parameter. Notably, “direct” FeN_4_ defects expanded the unit cell by approximately 0.1 Å up to 14.89 Å, while “tilted” FeN_4_ moieties contracted the unit cell by approximately 0.08 Å down to 14.71 Å, with respect to the undoped zigzag nanoribbon model (Figure 1), with an initial unit cell parameter of 14.79 Å. This methodology was not applied to AGNR models, as there is no effect of strain on spin moments on the non-magnetic armchair models. Discussing first the FeN_4_-ZGNR structures, it is worth noting that the spin distribution on the carbon edges was not affected by the in-plane defects in the baseline A1, B, and B1 locations. The total magnetic moment of these structures was equal to the magnetic moment at the Fe 3d electrons, because of the opposite spin directions of edge carbons that cancel when integrating the spin density over all atoms in the unit cell. The situation changed for the near-edge (C1) and on-edge (C) FeN_4_ locations, for which the carbon spin moments were quenched. Even more, not only did the spin density at each carbon drop down, but the sign of the spin moment also changed from −0.20 $\mu_{B}$ (B1 in Figure 4a) to 0.02 $\mu_{B}$ (from B1 to C in Figure 4a). Consequently, the near-edge FeN_4_-ZGNR entire structure became non-magnetic because the Fe spin-down moment cancelled the spin-up moment of the carbons at the opposite edge. On the contrary, the magnetic moment of the on-edge FeN_4_-ZGNR structure (FeN_4_ centered at C in Figure 4a) increased up to 4 ${\;\mu }_{B}$ (Table 1), resulting mainly from the parallel spin moments on the iron and on the opposite edge carbons. Interestingly, the spin quenching of the carbons at the edge close to the FeN_4_ site did not affect the absolute value of the Fe spin moment. Previously studied N-doped ZGNRs edges were established as magnetic [26], different from our findings for FeN_4_-doped ZGNR edges. Other DFT studies have reported on the spin suppression of edge carbon sites in Ti-, Pt-, and Au-doped ZGNR edges [52,53,54].

**Figure 5:**
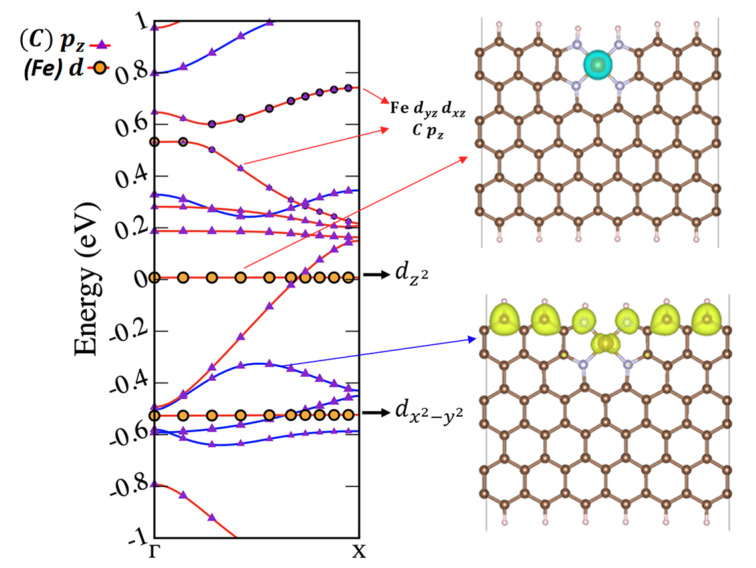
Charge-density decomposition of the sub-bands in the at-edge FeN_4_-ZGNR, showing the fat bands for the states labelled as C p_z_ and Fe 3d orbitals (**left**) side. The Fermi level in the sub-band diagram is set to zero. Spin-down (turquoise color) Fe $3d_{z^{2}}$ charge density is plotted in the upper (**right**) panel and the spin-up (yellow) Cp_z_ charge-density distribution is plotted in the right down panel.

The band structures depicted in Figure 4b also exhibited sensitivity to the FeN_4_ location, which was especially noticeable in the spin-down channel (red curves in Figure 4b). Localized flat-band characteristics due to $d_{x^{2}-y^{2}}$ , $d_{xy}$ , and $d_{z^{2}}$ states were observed in the band structures in all FeN_4_-ZGNRs, as shown by the blue and green arrows pointing to these d-bands in Figure 4b. The unoccupied $d_{z^{2}}$ localized band (pointed by a blue arrow in Figure 4b) shifted downward the Fermi level when moving the FeN_4_ defect from A1 to B1 to C1. The $d_{z^{2}}$ unoccupied band localized at energy levels of 0.34, 0.21, and 0.01 eV in the A1, B1, and C1 configurations, respectively. The contributions of $d_{yz}$ and $d_{xz}$ in the bands were intertwined with the p_z_ orbital contribution from carbon. This results in hybridization between the Cp_z_ and Fe $d_{xz}$ / $d_{yz}$ orbitals, as demonstrated for the C1 FeN_4_-ZGNR structure in Figure 5.

As the iron dopant was shifted towards the zigzag ribbon edge, the spin-down band gap diminished and closed entirely in the on-edge FeN_4_-ZGNR configuration. Consequently, the band structure became half-metallic, displaying a metallic character along the spin-down channel and a semi-conducting character with a 0.57 eV band gap along the spin-up channel (see Figure 4b, last column). Pt-doping at the ZGNR edges was also found to promote the half-metallic state [52]. The charge-density decomposition, presented in fat band in Figure 5, revealed that the half-metallic character was due to the spin-down Fe dz^2^ electrons at the Fermi level (the spin-density in the upper-right panel in Figure 5) and the Cp_z_ electrons, crossing the Fermi level. The valence band was formed by the occupied p_z_ electrons with opposite spins and the Fe $d_{x^{2}-y^{2}}$ electrons with spin down. The Fe3 $d_{yz}$ and 3 $d_{xz}$ orbitals were in the conduction bands. Assuming the validity of the semi-empirical rule correlating the smaller distance of the center of the 3d band of transition-metal surfaces with their enhanced catalytic activity [53] holds also for the half-metallic FeN_4_-doped graphene, a high reactivity could be suggested for the on-edge FeN_4_-ZGNRs, due to the 0.01 eV spacing between Fermi level and the center of the 3d band (see Figure 5).

The total spin moment in the doped structures with armchair termination was carried out essentially by Fe3d electrons (see Figure 4c for “direct” oriented defects and Figure S3 for “tilted” oriented defects), regardless of the location of the FeN_4_ defect. This result differs from the previously reported magnetization of AGNR edges due to doping with isolated transition metal and nitrogen atoms [21]. Nevertheless, the FeN_4_ dopants did not cause spin polarization of the armchair carbons, the band structure in the “direct” FeN_4_-dopants changes from semi-conducting in the in-plane-doped (position A) AGNR to half-metallic in the near- and on-edge-doped structures (see Figure 4d). For comparison, a band gap of 0.2 eV was calculated for the pristine AGNR, shown in Figure S4. The changes from semi-conducting to half-metallic states were not found for the “tilted” FeN_4_-AGNRs as reported in Figure S3. It follows that the FeN_4_ dopant orientation played a role on the AGNR band-structure features, but did not polarize the spin states.

The validity of the above discussed models and results was verified for a cell with a double length along the periodicity direction and integrating two FeN_4_ sites, the (2 × 1 × 1) supercell model, of in-plane “direct” FeN_4_-ZGNRs and “tilted” FeN_4_-AGNRs. This allowed also to explore the anti-parallel orientation of the spin moments on the two Fe sites, thus the AFM doped zigzag ribbons. Except the changes in the total magnetic moment due to the presence of two FeN_4_ defects in the supercell, the charge-densities and band-diagrams in Figure S5 (FeN_4_-ZGNRs) and Figure S6 (FeN_4_-AGNRs) show the same characteristics as those obtained for the respective unit cells, discussed in detail above. Moreover, considering two FeN_4_ dopants on the opposite ZGNR edges (see Figure S7) or two FeN_4_ on-edge defects at the same edge in a (2 × 1 × 1) supercell in Figure S8 always resulted in spin-quenching of the edge carbons, which is demonstrated with the nearly zero magnetic moments per carbon atom as reported in the Figure S8.

### 2.4. Discussion: Implication for Catalysis

Our results demonstrate that the coupling between iron magnetic moment and the magnetic moments of carbon atoms at the graphene edges significantly modifies the electronic structure of the materials depending on three factors: (i) graphene edge-geometries, i.e., zigzag or armchair; (ii) location of FeN_4_ in the graphene nanoribbon; and (iii) orientation of FeN_4_ with respect to the graphene edges (“direct” or “tilted”). This suggests that different possibilities exist for tuning the electronic characteristics of FeN_4_-graphene, which has been recognized as necessary for improving their catalytic activity in ORR [48].

Discussing the property variations induced by the presence of FeN_4_ at the edges, we first noted that the spin-unpolarized (non-magnetic) carbon states in the armchair terminated GNRs remained as such also when interacting with the FeN_4_ defects at the edges, but the FeN_4_-AGNR electronic structure changed from semi-conducting for the FeN_4_ integrated in-plane of AGNR to half-metallic for the “direct” FeN_4_ integrated at the edge (Figure 4d). Half-metallicity is revealed also by the band structure of the edge-doped ZGNRs (Figure 4b, last column). The spin suppression caused by the introduction of an FeN_4_ moiety at the edge of the ZGNR makes its electronic structure comparable with the spin-unpolarized AGNR edges, therefore suppressing the difference in the electronic properties between AGNR and ZGNR terminations. This suggests that the at-edge location of the FeN_4_ active site plays a preponderant role over the edge geometries in the electronic structure. Half-metallic electronic structure was found to be at the origin of the highest catalytic activity in FeN_4_-graphene for ORR [48]. We can therefore anticipate that edge-doped FeN_4_-GNRs would be highly catalytically active in ORR. Adding this to the highest thermodynamic stability of edge-doped ZGNR and AGNR (see Figure 2: points C1, left hand side and point D1, right hand side), edge-doped FeN_4_ graphene nanoribbons can be envisioned as the most promising candidates for ORR catalysis. Moreover, since the edge doping removes the effect of the specific geometrical termination on the graphene electronic structures, we can also extrapolate the validity of our findings to the pyrolyzed powder FeN_x_C_y_ catalysts, regardless of the geometrical organizations of their edge carbons.

Furthermore, the spin-down dz^2^ electrons in the at-edge FeN_4_-ZGNRs are practically localized at the Fermi level (see Figure 5), which would enhance their interaction with the spin-polarized πp* orbital of O_2_ (the ground state of O_2_ is a triplet spin state) or the doublet spin-state of OH*. The increase of spin asymmetry (the difference in the population of spin-up and spin-down states) in the band structure of MeN_4_-graphene catalysts was also suggested to improve the catalytic activity in the CO oxidation reaction [54]. Following the evolution of the band diagrams in Figure 4b,d, we observed that moving FeN_4_ from the in-plane to the edge resulted in increasing the differences between the populations of spin-down and spin-up channels close to the Fermi level, thus highlighting again the edge-doped FeN_4_ graphene GNRs as potentially highly active catalysts. Furthermore, the interactions between Cp_z_ and Fe3d magnetic moments can significantly modify the total magnetic moment of the entire FeN_4_-graphene material, as demonstrated in Table 1. The spin polarization of the edge carbons in the various FeN_4_-graphene models need to be considered in addition to the Fe spin density in the DFT calculations when accessing the magnetic properties of the active FeN_4_ sites.

## 3. Materials and Methods

Computational Details

All DFT calculations were performed using the Vienna Ab Initio Simulation Package [55,56]. The interactions between electron and nuclei were described within the framework of PAW formalism. The exchange–correlation energy was calculated within the Perdew, Burke, and Ernzerhof formulation of the generalized-gradient approximation (GGA-PBE) [57,58]. After the extensive test calculations for the total energy convergence, the kinetic energy cut-off for plane wave expansion was set to 500 eV. To simulate the periodic graphene nanoribbons (GNRs), the distance between the graphene layers was maintained at 15 Å for edge-edge and layer-layer distances. Structural relaxation is performed with a converge criteria of 1 × 10^−2^ eV/Å on force and a Γ-centered k-point grid of 15 × 1 × 1 for ZGNR model and 1 × 15 × 1 for AGNR model. Increasing the k-points up to 20 along the periodicity direction did not significantly change the energy values, confirming the adequacy of the chosen grid. An electron self-consistency loop was performed with accurate convergence of the magnetic state by assigning initial spin moments to each atom. We consistently employed the Fermi–Dirac smearing method across all cases, setting the smearing parameter to 0.03. The spin moment distribution of the FeN_4_ motif embedded GNRs models was calculated using the Bader analysis as described in ref. [59].

To study the formation of the FeN_4_ active sites, the computed formation energies $E_{f}$ for Fe-N_4_-GNR was based on the following equation

(1) $$E_{f}=\left((E_{Fe-N_{4}-GNR}+\left(\mu_{C}\times N_{subtracted}\right)\right)-\left(E_{GNR}+(\mu_{N}\times N_{substituted})+E_{Fe})\right)$$

where $E_{Fe-N_{4}-GNR}$ is the total energy of the Fe-N4 substituted model; $\mu_{C}$ is the chemical potential of carbon, defined as the total energy of graphene per carbon atom; $E_{GNR}$ is the total energy of pristine (perfect) GNR; $\mu_{N}$ is the chemical potential of nitrogen, taken as one-half of the total energy of the N_2_ molecule in the gas phase, and $E_{Fe}$ is the energy of the isolated Fe atom in the gas phase. In the above equation, the number of subtracted carbon atoms was six (two of them come from double carbon vacancy and the remaining four carbon atoms are subtracted to be replaced by nitrogen) and the number of substituted nitrogen atoms was four. Throughout this article, a lower formation energy signifies a higher probability of FeN_4_ formation on a selected position.

Fe binding energy was calculated as

(2) $$E_{B}=\left((E_{Fe-N_{4}-GNR}\right)-\left(E_{N4-GNR}+E_{Fe})\right)$$

The binding energies ( $E_{B}$ ) were defined as the difference between the energy of the Fe atom and N4 in the substitutional position of the GNRs and the energy of the reconstructed with double vacancy and substituted 4 N atoms ( $E_{N4-GNR}$ ) plus the energy of isolated Fe atom ( $E_{Fe}$ ).

The different initial spin moments on the two edges were considered to obtain the energy difference between the AFM and FM configurations for each doped ZGNR; however, unit cells with a single FeN_4_ motif allowed only for ferromagnetic solution. Thus, we doubled the unit cell along the x direction (2 × 1 × 1) to explore the energetically competing antiferromagnetic spin state.

Spin-density distribution analysis was performed using a two-phase computational approach. Initially, charge-density calculations were performed for both core and valence electron shells, and these densities were subsequently integrated to yield the total charge density. Magnetic charge density was derived by subtracting the spin-down charge density from the spin-up charge density. The final phase involved Bader analysis, which partitions the magnetic charge density with reference to the total charge density. The partial charge densities were calculated by taking into account all k-points. Subsequently, using a post-processing bash script, the spin-up and spin-down charge densities were separated from these partial charge densities within the fat-band profile.

## 4. Conclusions

In this study, we present an evaluation of the thermodynamic stability, electronic, and magnetic properties as a function of the position and orientation of FeN_4_, covalently integrated into graphene nanoribbons with zigzag and armchair terminations in view of their potential applications as single-atom electrocatalysts. The DFT calculations with periodic boundary conditions were employed. Our results demonstrate that the covalent integration of FeN_4_ defects in both ZGNR and AGNR results in thermodynamically stable materials independent of the doping location. Nevertheless, the differences in the binding and formation energies oscillates within 15%; at-edge doped GNRs are the most energetically stable structures.

The spin-moment distribution in zigzag graphene nanoribbons that integrate FeN_4_ is not determined only by the spin polarization of 3d electrons, but also by the spin polarization of the Cp_z_ electrons. The spin-polarized edge carbons interact with Fe3d spin-polarized states if FeN_4_ is located near or at the edge of the ZGNR, which results in suppression of the spin moments of edge carbons. In the latter cases, the Cp_z_ electrons change their spin orientations, but the Fe3d electrons preserve their spin moments. Consequently, the total magnetic moment of the FeN_4_-ZGNRs varies according to the spin orientations on the carbons at the opposite edge and at iron. The spin-unpolarized states of the edge carbons in AGNR remain unaffected by the FeN_4_ spin moments.

The spin quench at edge carbons is manifested with the reduction or removal of edge electronic states from Fermi level in the band gap. The FeN_4_ at-edge location transforms the electronic structures to half-metallic and increases the spin asymmetry in both ZGNR and AGNR; therefore, integrating FeN_4_ at the edges removes the effect of edge geometries on the electronic properties of the catalyst. This suggests that the location of the FeN_4_ active sites will be determinant not only for the catalyst stability, but also for its reactivity, being recognized as highly dependent on the FeN_4_-graphene magnetic and electronic structures. Since the edge geometries play a minor or non-role in the electronic structure of at-edge FeN_4_-graphene, FeN_4_-GNRs can be considered as relevant models for describing the magnetic and electronic properties in relation to the reactivity of pyrolyzed Fe-N-C catalysts.

## Figures and Tables

**Table 1:** Total magnetic moment (μ_Total_) and Fe magnetic moment (μ_Fe_) in ${\;\mu }_{B}$ of FeN_4_-ZGNRs with initial AFM spin ordering at the edges.

FeN_4_ Position	A1	B	B1	C	C1
μ_Total_	−2.00	2.02	−1.97	−0.09	4.00
μ_Fe_	−2.04	1.99	−2.10	−2.02	2.12

## Data Availability

Data are contained within the article and supplementary materials.

## Supplementary Materials

The following supporting information can be downloaded at: www.mdpi.com/xxx/s1, Figure S1: Spin density distributions and sub-band diagrams of FM and AFM ordered ZGNRs; Figure S2: Charge density composition of AFM edges of the undoped ZGNR; Figure S3: Spin-density and sub-band structures in “tilted” FeN_4_-AGNR structures; Figure S4: The lattice structure and sub-band diagram of AGNR; Figure S5: Spin density and band diagram of FM and AFM ordered (2 × 1 × 1) supercell of FeN_4_-ZGNRs; Figure S6: Spin density and band diagram of FM and AFM ordered (2 × 1 × 1) supercells of FeN4-AGNR; Figure S7: Spin density FM and AFM ordered at-edge FeN_4_-ZGNRs; Figure S8: Spin density of at-edge FeN_4_ ZGNR (supercell lattice [2 × 1 × 1]).
