# Supplementary material for: Exploring Spin Distribution and Electronic Properties in FeN4-Graphene Catalysts with Edge Terminations
Source: Molecules. 2024 Jan 18;29(2):0. doi: 10.3390/molecules29020479 (PMC11154451; doi:10.3390/molecules29020479)
Supplement: Supplementary file 1 [file molecules-29-00479-s001.zip › molecules-2813789-supplementary.pdf]

Exploring Spin Distribution and Electronic Properties in FeN_4-Graphene Catalysts with Edge Terminations

Ismail Can Oguz, Frederic Jaouen and Tzonka Mineva *

ICGM, Univ. Montpellier, 34293 Montpellier, France; i.c.oguz@differ.nl (I.C.O.); frederic.jaouen@umontpellier.fr (F.J.)
* Correspondence: tzonka.mineva@enscm.fr

Supporting Information

Supporting Figures

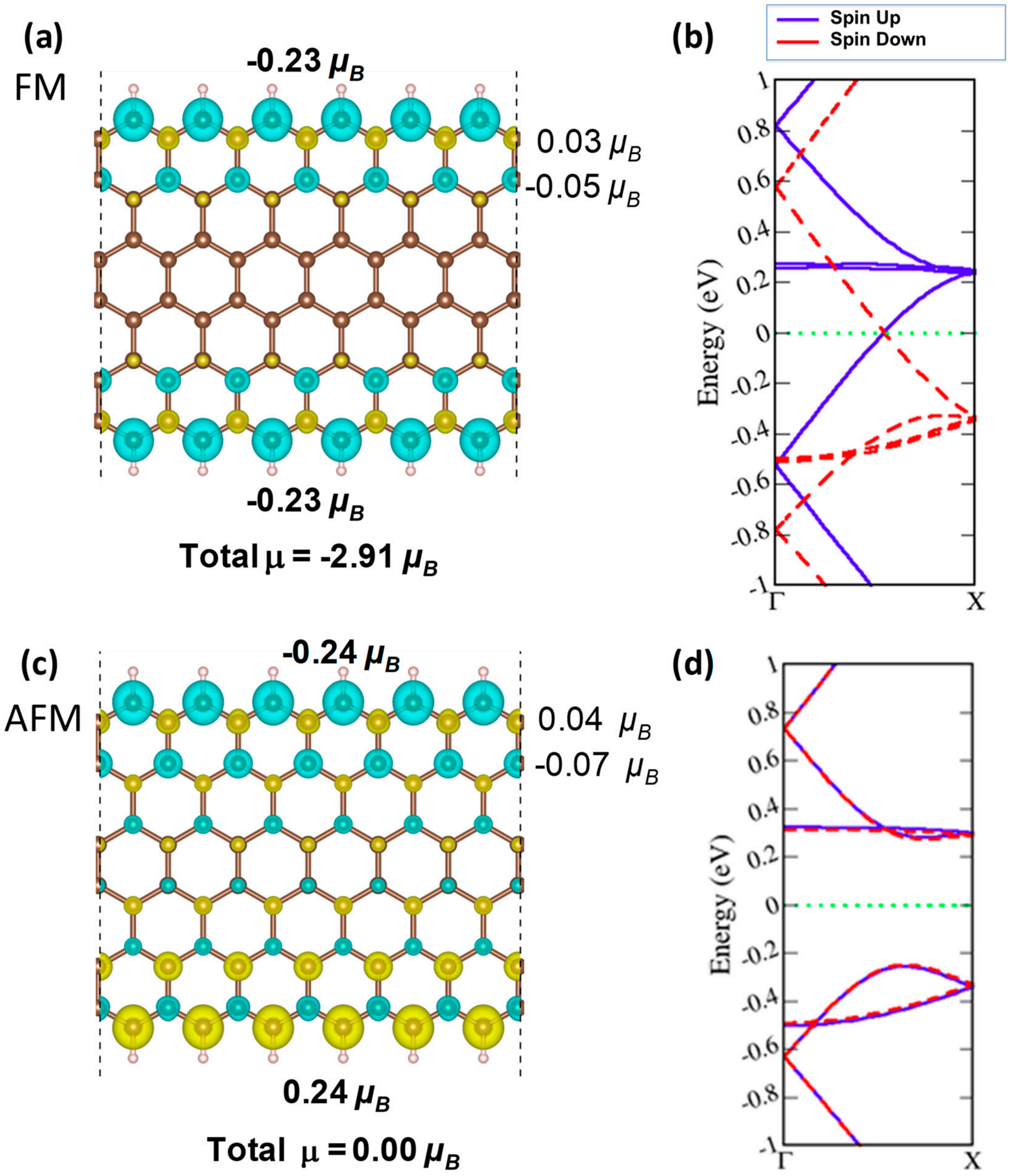

Figure S1 Spin density distributions (a, c) and sub-band diagrams (b, d) of FM (top) and AFM (bottom) ordered ZGNRs. Yellow and turquois color represent the spin up and spin down distribution over lattice structure. Blue and dashed red color represent the spin up and spin down states in band diagram. The metallic character is found for FM structure and semiconducting character is found for AFM structure with p_z states dominating around the Fermi level.

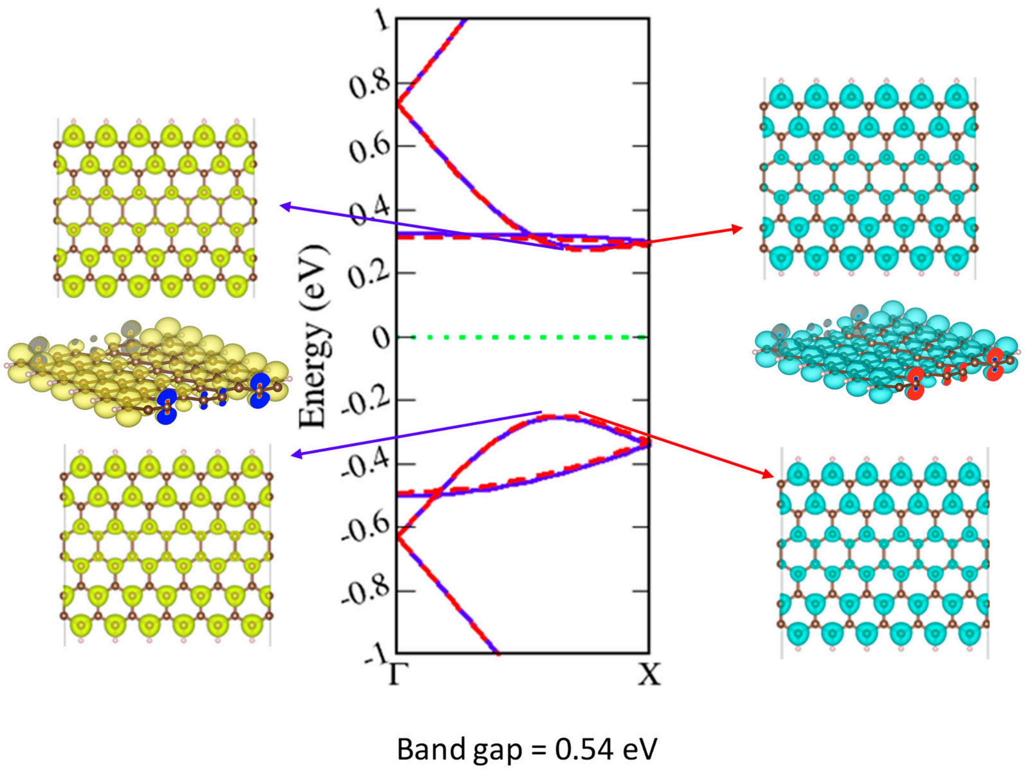

Figure S2. Charge density composition of the spin-up (left) and spin-down (right) bands, plotted in the middle panel for the case of AFM edges of the undoped ZGNR. Yellow and turquois color represent the spin up and spin down distribution over lattice structure. The Fermi energy is set to zero. The p_z carbon orbitals are better illustrated in the perpendicular plane to the ZGNR plane.

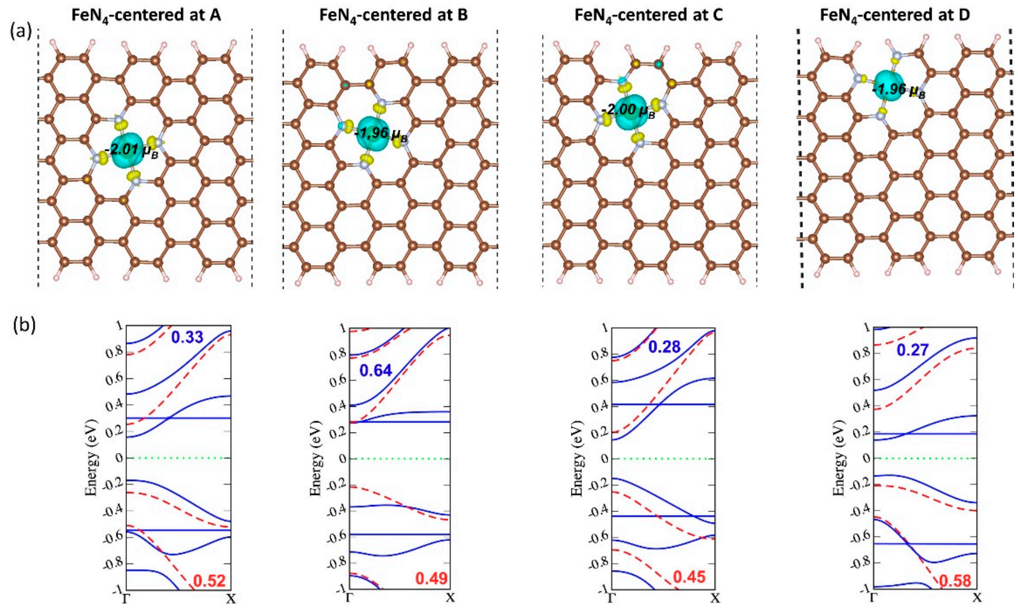

Figure S3. Spin-density (a) and sub-band structures (b) in "tilted" FeN4-AGNR structures. The bands of spin-up channels are in blue and the bands of spin-down channels are in red. Yellow and turquois color represent the spin up and spin down distribution over lattice structure. The band-gaps, reported in the band diagrams are in eV.

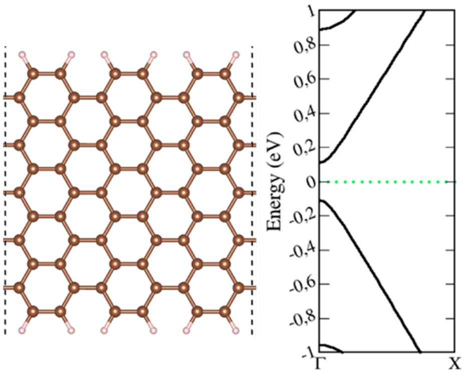

Figure S4. The lattice structure (left) and sub-band diagram of AGNR (right).

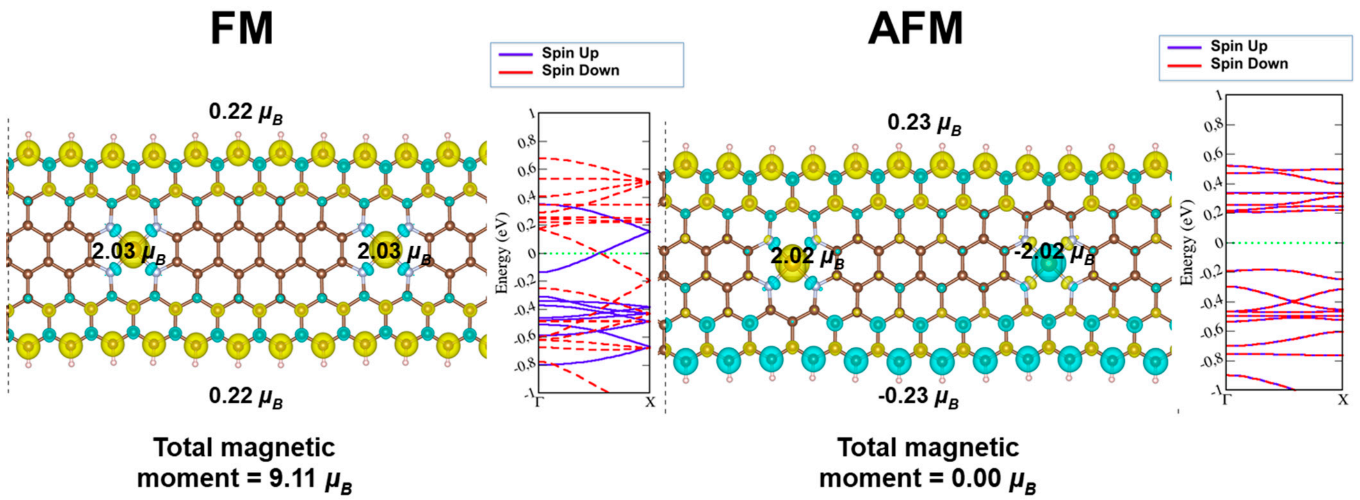

Figure S5 Spin density and band diagram of FM and AFM ordered (2x1x1) supercell of FeN_4-ZGNRs. Yellow and turquois color represent the spin up and spin down distribution over the lattice structure. Blue and dashed red color represent the spin up and spin down states in the band diagrams.

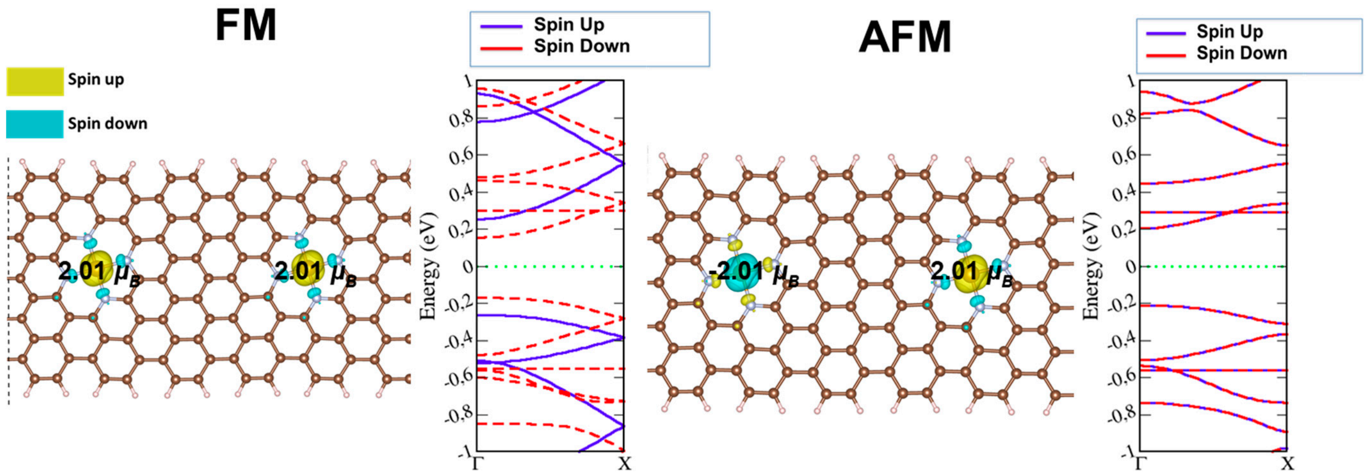

Figure S6 Spin density and band diagram of FM and AFM ordered (2x1x1) supercells of FeN4-AGNR. Yellow and turquois color represent, respectively, the spin up and spin down distribution over lattice structure. Blue and dashed red color represent, respectively, the spin up and spin down states in the band diagrams.

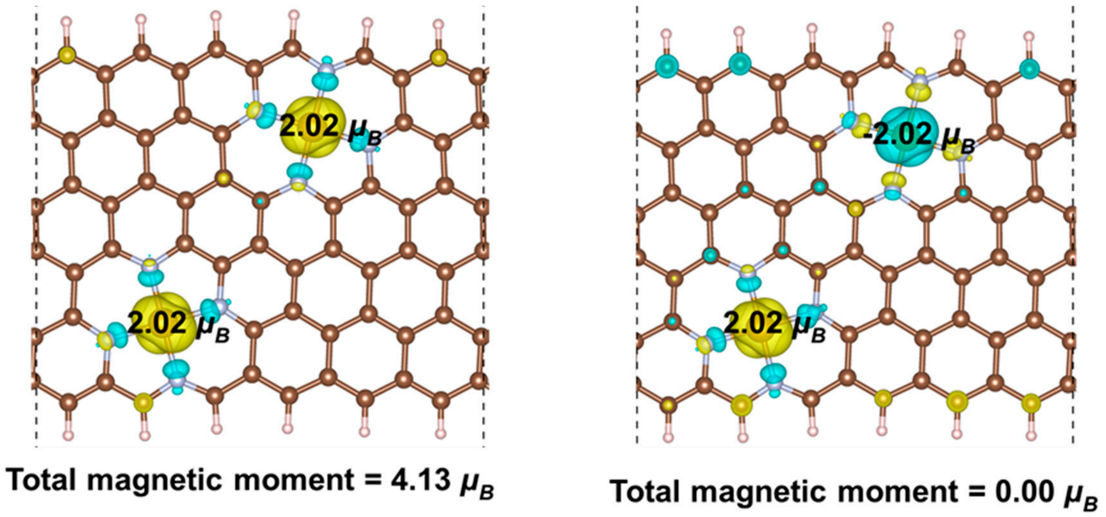

Figure S7 Spin density FM (left) and AFM (right) ordered on-edge FeN4 - ZGNR. Yellow and turquois color represent, respectively, the spin up and spin down distribution over the lattice structure.

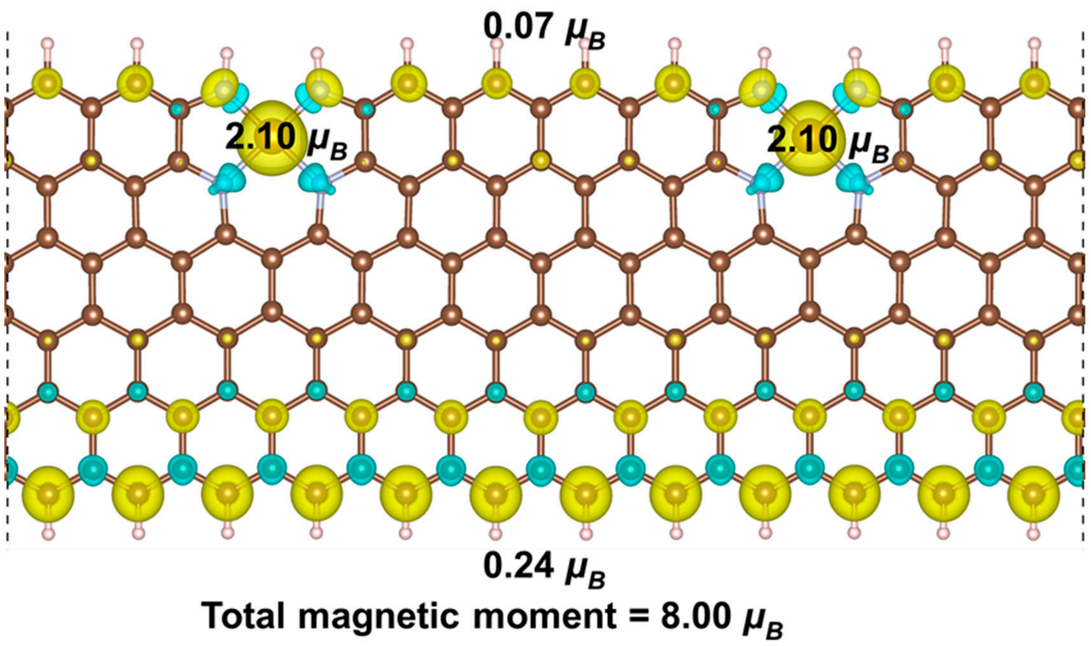

Figure S8. Spin density of at-edge FeN_4 ZGNR (supercell lattice [2x1x1]). Yellow and turquois color represent the spin up and spin down distribution over lattice structure.
